# Subclinical Atherosclerosis in Alopecia Areata: Usefulness of Arterial Ultrasound for Disease Diagnosis and Analysis of Its Relationship with Cardiometabolic Parameters

**DOI:** 10.3390/jcm13144264

**Published:** 2024-07-22

**Authors:** Patricia Burgos-Blasco, Alvaro Gonzalez-Cantero, Angela Hermosa-Gelbard, Juan Jiménez-Cahue, Diego Buendía-Castaño, Emilio Berna-Rico, Carlota Abbad-Jaime de Aragon, Sergio Vañó-Galván, David Saceda-Corralo

**Affiliations:** 1 Servicio de Dermatología, Hospital Universitario Ramón y Cajal, Instituto Ramón y Cajal de Investigación Sanitaria (IRYCIS), Carretera Colmenar Viejo km 9.100, 28034 Madrid, Spain; ahermosagelbard@gmail.com (A.H.-G.); jjimenezc92@gmail.com (J.J.-C.); dbuendiac@gmail.com (D.B.-C.); emilioberna2a@gmail.com (E.B.-R.); carlotababbad@gmail.com (C.A.-J.d.A.); drsergiovano@gmail.com (S.V.-G.); d.saceda@gmail.com (D.S.-C.); 2 Hair Disorders Unit, Grupo Pedro Jaén, 28006 Madrid, Spain; 3 Faculty of Medicine, Universidad Francisco de Vitoria, 28223 Pozuelo de Alarcón, Spain; 4 Departamento de Biología de Sistemas, Facultad de Medicina, Universidad de Alcalá, 28801 Alcalá de Henares, Spain

**Keywords:** hair loss, cardiovascular risk factor, ultrasound, autoimmune-induced hair loss

## Abstract

**Background/Objectives:** Chronic systemic inflammation is a risk factor that increases the development of atherosclerosis and predisposes to cardiovascular diseases (CVDs). The systemic inflammatory profile of alopecia areata (AA) regarding IFNγ and Th1 cytokine dysregulation has previously been described, suggesting an increased incidence of CVDs in this population. No previous studies investigated the possible relationship between atherosclerosis and AA by cardiovascular imaging techniques. To determine the prevalence, distribution and burden of subclinical atherosclerosis in AA. Methods: We conducted a case–control study in 62 participants, including 31 patients with severe AA (SALT > 75) and 31 healthy controls, matched for age, sex and body mass index (BMI). The participants underwent a detailed history assessment and were subjected to the measurement of weight, height, abdominal circumference and blood pressure. A fasting blood sample was also collected. Subclinical atherosclerosis was evaluated by ultrasonography of the bilateral femoral and carotid arteries. Results: The AA patients had an increased prevalence of subclinical atherosclerosis (54.7%) compared to the healthy controls (22.6%, *p* = 0.010). The prevalence of atheroma plaques was significantly higher in the carotid arteries (41.90% vs. 12.9%, *p* = 0.009), while no significant differences were found in femoral plaque prevalence. The AA patients with atherosclerotic plaques were older (*p* < 0.001) and had a longer time since AA diagnosis (*p* = 0.11) and increased serum levels of glycated hemoglobin (*p* = 0.029) and triglycerides (*p* = 0.009). In a regression analysis, duration of disease and neutrophil/lymphocyte ratio were the main predictors of atherosclerosis. Conclusions: AA patients have an increased prevalence of carotid subclinical atherosclerosis. The duration of AA, systemic inflammation and insulin resistance appear to play a role in the development of subclinical atherosclerosis in this population.

## 1. Introduction

Alopecia areata (AA) is a chronic immune-mediated disease that most frequently presents as well demarcated areas of sudden and asymptomatic hair loss on the scalp [1]. It is, however, a complex disease with increasing prevalence and great clinical variety that can affect any surface with hair follicles [2]. Although traditionally considered an organ-specific disease limited to the hair follicle, its systemic nature has recently been established [3]. The key role of systemic immunity is supported by an increased risk of autoimmune diseases, a higher prevalence of autoantibodies and the use of immunosuppressants as the basis of AA treatment [4,5,6].

AA is considered a multifactorial disease in which environmental factors act on genetically predisposed individuals, triggering the collapse of the hair follicle’s immune privilege, which is then maintained through a local increase in IFNγ and IL-15 via the JAK/STAT pathway [7,8]. This peribulbar inflammatory cascade subsequently disrupts hair follicle cycling and leads to a premature entry into the telogen phase, causing the clinical phenotype of AA [9]. In addition, systemic inflammatory changes have been detected in AA patients, involving both innate immunity, with increased serum levels of IL-6, IL-8 and IFNγ, and the adaptive immune system, with a systemic dysregulation of Th1 cytokines. These alterations have been found to be more prominent in patients with a greater severity of the disease and a longer time since AA diagnosis [3,10,11]. 

Previous studies reported that inflammatory dysregulation in patients with immune-mediated diseases is associated with a higher risk of cardiovascular disease (CVD). Systemic inflammation, when maintained chronically in time, can become a risk factor that contributes, together with traditional cardiovascular risk factors (CVRFs), to the development of accelerated atherosclerosis [12,13]. Atherosclerosis is even considered a chronic inflammatory disorder of the arterial wall, in which endothelial dysfunction and hemodynamic damage in areas of turbulent blood flow favor the expression of adhesion molecules and local inflammatory cytokines (IL-6 and IL-8), triggering lipid deposition, smooth muscle proliferation and the formation of an atherosclerotic plaque [14]. The importance of atherosclerosis relies on it being the base of CVD. It is well established that increased inflammation may compromise the stability of existing atherosclerotic plaques, causing plaque rupture or thrombosis, which may result in blood vessel occlusion and acute cardiovascular events [15].

It has been hypothesized that patients with AA may therefore be at great risk of subclinical atherosclerosis and CVD. However, the published data are scarce, and an association is yet to be proven. The aim of this study was to determine the prevalence, territorial distribution and burden of subclinical atherosclerosis in AA patients compared to healthy individuals.

## 2. Materials and Methods 

### 2.1. Study Design

We conducted a case–control study at the Hair Disorders Unit of the University Hospital Ramón y Cajal in Madrid. The study was approved by the local research ethics committee and conducted in accordance with the principles of the Declaration of Helsinki. All participants provided written informed consent. All participants were consecutively and voluntarily recruited from April 2021 to October 2022. 

### 2.2. Inclusion and Exclusion Criteria

The inclusion criteria were a diagnosis of AA with more than 75% of scalp involvement (SALT > 75), age over 18 years and the absence of systemic treatment for AA (4 weeks for standard immunosuppressive treatment and 12 weeks for anti-JAK therapy). The diagnosis of AA and SALT evaluation were performed by board-certified dermatologists. The healthy controls were randomly recruited from among patients attending the Dermatology Department with non-inflammatory dermatological diagnoses and from among hospital personnel. The inclusion criteria for this group were age 18 years or older and the absence of a personal or family history of AA or of any autoimmune-induced alopecia. AA patients and healthy controls were matched 1:1 for sex, age and body mass index (BMI).

Exclusion criteria, for both cases and healthy controls, were a personal history of cardiovascular or cerebrovascular diseases or the presence of other chronic inflammatory conditions, including psoriasis, atopic dermatitis and rheumatoid arthritis.

### 2.3. Clinical, Antrhopometrical and Laboratory Variables

All participants underwent a full medical history assessment, with data regarding sex, age, smoking habits, diagnosis of CVRFs (hypertension, dyslipidemia, diabetes and metabolic syndrome) and drug intake. In the AA group, SALT and time since the first episode of AA were also noted. 

A physical examination was performed recording weight, height and abdominal circumference measured in the standing position using a flexible tape at the midpoint between the lowest rib and the iliac crest. Blood pressure was then determined as the mean of three consecutive measurements taken 5 min apart with an automatic sphygmomanometer (M6 AC, OMRON Healthcare Co. Ltd., Kyoto, Japan).

Blood samples were drawn after at least 8 h of fasting. Blood parameters were measured using standard automated techniques and consisted of a complete blood count and of the serum levels of triglycerides, total cholesterol, high-density lipoprotein cholesterol (HDL-C), low-density lipoprotein cholesterol (LDL-C), liver enzymes (AST, ALT, GGT, alkaline phosphatase), C-reactive protein (CRP), albumin, glucose, glycosylated hemoglobin and insulin. Regarding indexes, the neutrophil-to-lymphocyte ratio (NLR) was calculated as a simple ratio between the neutrophil and the lymphocyte counts measured in the peripheral blood [16], and the calculation formula for the triglyceride–glucose index (TyG) was as follows: TyG = Ln [fasting triglycerides (mg/dL) × fasting blood glucose (mg/dL)/2] [17].

### 2.4. Ultrasound Study

**Figure 1:**
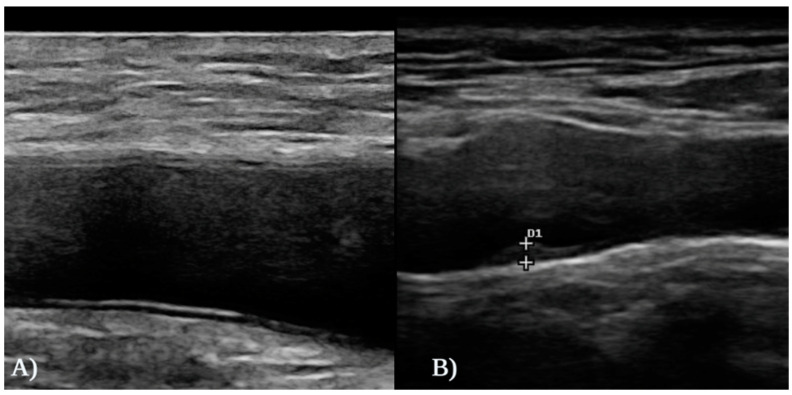
(**A**) Ultrasound imaging of the right common femoral artery of a healthy control without subclinical atherosclerosis. (**B**) Atherosclerotic plaque of 1.8 mm detected by ultrasound of the left carotid artery in a patient with AA.

All subjects underwent a systematic carotid and femoral ultrasound examination with a Clarius^®^ Ultra-High Frequency Linear Scanner (L20 HD3, Vancouver, BC, Canada). The ultrasound examination was performed bilaterally in the transversal and longitudinal planes as previously described, covering all the territory from the supraclavicular fossa to the submandibular angle and the 20 mm segment proximal to the common femoral artery bifurcation into superficial and deep femoral arteries [18,19]. An atherosclerotic plaque was defined as a focal structure protruding into the arterial lumen ≥ 0.5 mm or with a thickness ≥ 50% of the surrounding intimal layer, or with a diffuse thickening of ≥1.5 mm (Figure 1) [18]. The thickness of each atherosclerotic plaque was determined as the mean value of three measurements. The diagnosis of subclinical atherosclerosis was defined as the presence of at least one atherosclerotic plaque in any of the studied arteries. The subclinical atherosclerosis burden was assessed according to the number of vascular territories affected.

### 2.5. Statistical Analysis

Statistical analysis was performed using SPSS version 20.9 (SPSS, Chicago, IL, USA). The Kolmogorov–Smirnoff test was used to check the normality of the data distribution. Quantitative variables are expressed by their mean, alongside with their standard deviation (SD), while qualitative variables are expressed by absolute frequency and percentage. The variables were compared between the groups using the Pearson chi-square test or Fisher’s exact test for qualitative variables, and Student’s *t*-tests or Mann–Whitney *U* test for quantitative variables. Binary logistic regression analysis was performed to establish the most significant determinants of subclinical atherosclerosis. A *p*-value < 0.05 was considered significant for all the analyses performed.

## 3. Results

The study population comprised 62 participants, including 31 patients with AA and 31 healthy controls, matched 1:1 for sex, age and BMI. The mean age among was 45.0 ± 11.6 and 44.8 ± 12.2 years for AA patients and healthy subjects, respectively (*p* = 0.949). Within the AA patients, the time since AA diagnosis was 15.5 ± 12.0 years, with a mean SALT of 98.8 ± 3.4.

### 3.1. Clinical, Anthropometrical and Laboratory Results

Regarding the CVRFs, a significantly higher prevalence of dyslipidemia was found among the AA patients compared to the control group (70.97% vs. 42.86%, *p* = 0.029). No significant differences were found regarding age, sex, BMI, abdominal circumference, tobacco consumption or arterial hypertension. The anthropometrical and clinical data of the participants are displayed in Table 1. 

As for the biochemical data, the AA patients presented with significantly lower albumin levels (3.99 ± 0.3 vs. 4.55 ± 0.2, *p* = 0.031) and a significantly higher neutrophil count (3.92 ± 1.4 × 10^3^ vs. 3.12 ± 1.0 × 10^3^, *p* = 0.028). No other significant differences were found between the groups. The full biochemical data are shown in Table 2. 

### 3.2. Femoral and Carotid Atherosclerosis

**Figure 2:**
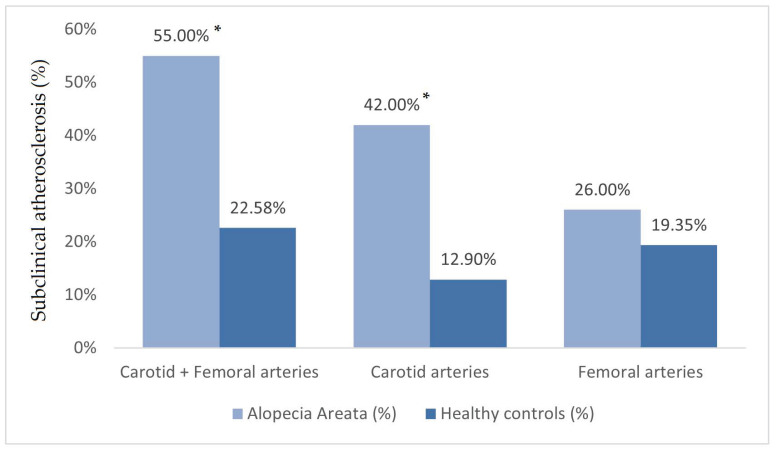
Percentage of participants with subclinical atherosclerosis and presence of atheroma plaques in carotid and femoral arteries in patients with AA and healthy controls. *: *p* < 0.05.

**Figure 3:**
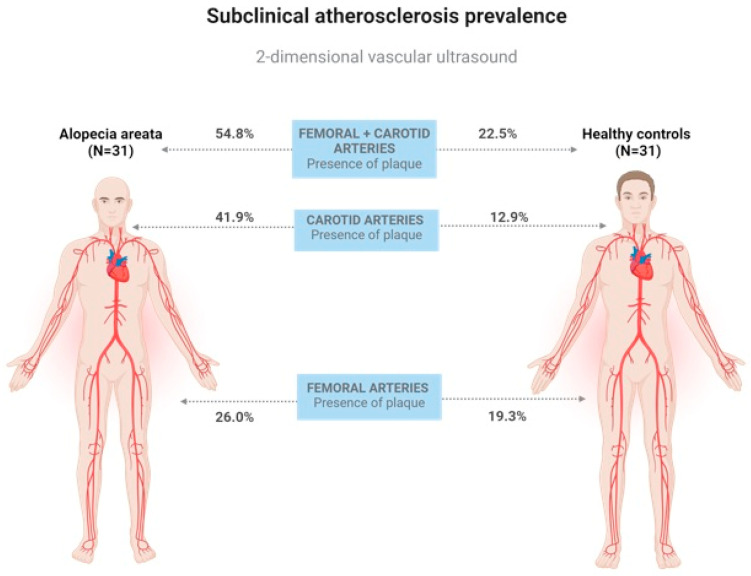
Prevalence of subclinical atherosclerosis in AA patients and controls measured with vascular ultrasound. Created with BioRender.com.

The prevalence of subclinical atherosclerosis was significantly higher in patients with AA than in healthy controls (54.84% vs. 22.58%, *p* = 0.010). Carotid plaque prevalence was significantly increased among the AA patients compared to the controls (*p* = 0.009). No differences were found regarding the prevalence of atherosclerosis plaques in the femoral territory (*p* = 0.543). The prevalence of subclinical atherosclerosis in the study population is exhibited in Figure 2 and Figure 3. 

Ten AA patients (32.3%) showed an involvement of at least two of the four territories studied, and four (12.9%) had an atherosclerotic burden of three or more territories. There were no significant differences between patients with AA and healthy controls regarding the atherosclerotic burden.

### 3.3. Relationship of Clinical, Anthropometrical and Laboratory Variables with Atherosclerosis

AA patients with subclinical atherosclerosis had a significantly higher age (*p* < 0.001), a longer time since AA diagnosis (*p* = 0.14), higher serum triglyceride levels (*p* = 0.017) and an increased percentage of serum glycated hemoglobin (*p* = 0.003) compared to AA patients without atherosclerotic plaques. The serum lymphocyte levels (*p* = 0.021) were lower in the former group compared to AA patients without subclinical atherosclerosis. Regarding the calculated indexes, significantly increased levels of NLR (*p* = 0.004) and TyG (*p* = 0.013) were observed in AA patients with femoral and/or carotid plaques. No other significant differences were found between the groups. The comparison of anthropometric, clinical and biochemical data between AA patients with and without subclinical atherosclerosis is shown in Table 3. 

### 3.4. Regresion Analysis

The stepwise logistic binary regression analysis revealed that the main variables that predicted the presence of subclinical atherosclerosis in AA patients were age (OR = 1.24; *p* = 0.008) and the neutrophil/lymphocyte ratio (OR = 18.029; *p* = 0.06). 

## 4. Discussion 

Immune-mediated diseases are recognized risk factors for the development of accelerated atherosclerotic CVDs. Chronic systemic inflammation, a defining feature of immune-mediated diseases, exerts a detrimental effect on the arterial endothelium, the innermost layer of cells lining the blood vessels. This persistent inflammatory state alters the endothelial function, resulting in impaired blood flow and increased adhesion of inflammatory cells to the arterial wall [12,13]. Patients with AA may also be predisposed to an increased risk of CVD, due the systemic inflammatory nature of this disease, although this is not yet well understood. For this purpose, we conducted the first study investigating subclinical atherosclerosis by imaging techniques in peripheral vascular territories in AA patients.

Regarding the prevalence of CVRFs, our study revealed a significantly higher prevalence of dyslipidemia in the AA patients than in the control group. These findings are similar to those of numerous population-based studies in patients with AA, which indicate that dyslipidemia is the second most frequent comorbidity in this subgroup [20,21]. It should be noted that, despite the fact that these studies support our finding of an increased risk of dyslipidemia, the reported prevalence of dyslipidemia among AA patients does not exceed 25%, a figure far from that obtained in this study. However, Nasimi et al. [22] showed a significant association between the diagnosis of dyslipidemia and the extension of AA, which could justify the high prevalence we obtained, given that only patients with a SALT > 75 were included. Although our AA patients showed a significantly higher prevalence of dyslipidemia, a known CVRF associated with atherosclerosis, the regression analysis did not reveal this CVRF as a possible predictor of subclinical atherosclerosis. This, together with the absence of differences in other traditional CVRFs between AA and controls, may give greater value to the ultrasound findings. 

There were no significant differences regarding other traditional CVRFs (abdominal obesity, tobacco consumption, hypertension). A higher prevalence of isolated diastolic hypertension was reflected in our series of AA patients, similar to what Karadag et al. [23] stated. However, although statistically significant, the differences found are probably not clinically relevant, given that isolated diastolic hypertension has not been shown to increase the risk of CVD [24].

Our results reflect a higher prevalence of subclinical atherosclerosis, after the ultrasound study of the carotid arteries, in AA patients than in the control group, with a similar prevalence of femoral atherosclerotic plaques (Figure 3). This differential impact in different territories may explain the higher incidence of cerebrovascular disease among patients with AA, without an increase in the risk of peripheral vascular disease or heart disease [20,25,26]. These findings are in accordance with studies in patients with rheumatoid arthritis, systemic lupus erythematosus and human immunodeficiency virus infection, reporting a higher pro-atherosclerotic effect of chronic inflammation in the carotid arteries [27,28]. In contrast, our results differ from those of studies performed in patients with psoriasis and in the general population, where the progression of atherosclerosis appeared to begin in the femoral territory [29,30].

**Figure 4:**
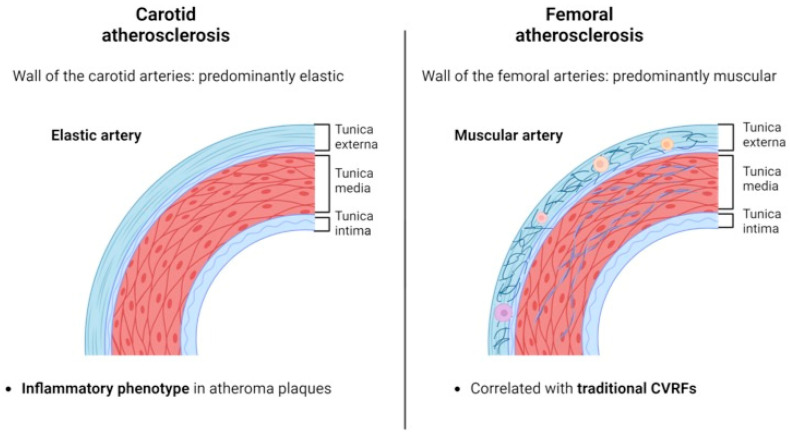
Arterial wall layer composition and differences between the carotid and femoral arteries. Created with BioRender.com.

The reason for the variable impact of inflammatory diseases in the development of atherosclerosis on the carotid and femoral arteries is poorly understood, although differences in the arterial wall may be involved (Figure 4). The femoral arteries have a greater muscular component similar to that of the walls of the coronary arteries, which results in a correlation between the presence of atherosclerotic plaques in these territories [31].

As shown previously in general-population studies, a diagnosis of femoral atherosclerosis is strongly associated with coronary artery disease [32]. Furthermore, the accumulation of traditional CVRFs is known to have a great atherogenic effect on the femoral and coronary territories [27,33]. The analysis of CVRFs revealed no significant differences between the control group and the patients with AA, excluding dyslipidemia. This observed low prevalence of traditional risk factors in the two groups might explain the similar prevalence of femoral atherosclerotic plaques detected among AA patients and healthy controls. 

On the other hand, the wall of the carotid arteries has a dominant elastic layer [31]. Interestingly, atherosclerosis at this level is less related to classic CVRFs, with plaque histological composition presenting a higher proportion of macrophages and inflammatory cells than in femoral atherosclerotic plaques [27,34]. Our findings showed that the AA patients who had subclinical atherosclerosis were older, with a longer time since diagnosis and with significantly higher insulin resistance and NLR. In fact, only the NLR and age could predict the presence of subclinical atherosclerosis. The NRL index is a well-known biomarker of inflammation that reflects the balance between acute and chronic inflammation (indicated by the neutrophil count) and adaptive immunity (indicated by the lymphocyte count). This marker has previously been studied in other inflammatory skin diseases, such as psoriasis and hidradenitis suppurativa, showing its possible utility as a subclinical tool for the screening of atherosclerosis and CVDs [35,36]. In our study, although not statistically significant, the NLR could be a marker to further explore in AA patients.

In all, these results suggest that systemic inflammation in AA plays a key role in the development of atherosclerosis, which could explain a higher prevalence of carotid atherosclerotic plaques in comparison with femoral involvement. Carotid ultrasound is therefore a noninvasive tool useful in the screening of patients with AA, in order to detect subclinical atherosclerosis and to identify those at risk of future cardiovascular events.

The study limitations include the small sample size and the restriction to patients with a great extension of alopecia (SALT > 75). This strict inclusion criterion was aimed to allow for only the inclusion of patients with severe disease and high systemic inflammatory dysregulation, which probably makes our results not applicable to all AA patients. Further studies with a larger sample size are required to validate our findings in moderate and mild cases of AA. The main study strength is the 1:1 matching between patients and controls for age, sex and BMI. 

## 5. Conclusions 

Patients with AA showed a higher prevalence of carotid subclinical atherosclerosis than healthy controls, with no differences regarding the femoral involvement. There was an increase in the prevalence of dyslipidemia among patients with AA, with no differences regarding other CVRFs. The duration of AA, systemic inflammation measured with the NLR and insulin resistance measured with the TyG may play a key role in the development of subclinical atherosclerosis, together with traditional CVRFs.

## Figures and Tables

**Table 1:** Anthropometrical and clinical data of patients with AA and healthy controls.

	Alopecia Areata N = 31	Healthy Controls N = 31	*p*
Sex (m/f)	12/19	12/19	NS
Age (years)	45.03 ± 11.6	44.84 ± 12.2	NS
Body mass index (kg/m^2^)	25.54 ± 3.9	25.79 ± 3.8	NS
Waist circumference	88.58 ± 12.6	89.68 ± 11.2	NS
Systolic blood pressure (mmHg)	124.44 ± 13.6	124.29 ± 13.1	NS
Diastolic blood pressure (mmHg)	83.18 ± 7.9	77.64 ± 8.3	0.017
Smoking (%)	21.42%	19.35%	NS
Hypertension (%)	70.97%	64.28%	NS
Dyslipidemia (%)	70.97%	42.86%	0.029
Time since AA diagnosis (years)	15.46 ± 12.0	-	-
SALT	98.75 ± 3.4	-	-

SALT, Severity of Alopecia Tool; NS, non-significant. Data expressed as % (qualitative variables) or as mean ± standard deviation (quantitative variables).

**Table 2:** Biochemical data of patients with AA and healthy controls.

	Alopecia Areata N = 31	Healthy Controls N = 31	*p*
Glucose (mg/dL)	90.36 ± 9.3	90.76 ± 6.9	NS
Albumin (mg/dL)	3.99 ± 0.3	4.55 ± 0.2	0.031
Cholesterol (mg/dL)	205.10 ± 44.2	191.29 ± 36.2	NS
HDL (mg/dL)	64.42 ± 24.3	61.59 ± 14.3	NS
LDL (mg/dL)	121.26 ± 31.6	115.37 ± 35.3	NS
Triglycerides (mg/dL)	95.39 ± 52.0	74.89 ± 27.6	NS
AST (U/L)	23.90 ± 15.6	21.29 ± 6.3	NS
ALT (U/L)	24.16 ± 21.3	20.54 ± 12.1	NS
GGT (U/L)	28.06 ± 22.8	19.04 ± 11.9	NS
Alkaline phosphatase (U/L)	70.68 ± 21.8	65.96 ± 30.5	NS
CRP (mg/dL)	3.70 ± 7.3	1.54 ± 1.2	NS
HbA1-c (%)	5.37 ± 0.3	5.26 ± 0.2	NS
Neutrophils (×10^3^/mL)	3.92 ± 1.4	3.12 ± 1.0	0.028
Lymphocytes (×10^3^ mL)	2.25 ± 0.7	2.15 ± 0.5	NS
Platelets (×10^3^/mL)	244.13 ± 55.9	237.38 ± 44.8	NS
NLR	1.86 ± 0.8	1.49 ± 0.4	NS
SII	463.60 ± 238.8	364.13 ± 136.4	NS
TyG	8.25 ± 0.5	8.04 ± 0.4	NS

CRP, C-reactive protein; NLR, neutrophil/lymphocyte ratio; SII, systemic inflammatory index; TyG, triglyceride–glucose index; NS, non-significant. Data expressed as mean ± standard deviation.

**Table 3:** Anthropometric, clinical and biochemical data in patients with AA according to the presence or absence of subclinical atherosclerosis.

	Alopecia Areata with Subclinical Atherosclerosis N = 17	Alopecia Areata without Subclinical Atherosclerosis N = 14	*p*
Sex (m/f)	4/13	8/6	NS
Age (years)	52.06 ± 7.8	44.84 ± 12.2	NS
Body mass index (kg/m^2^)	25.80 ± 4.3	36.50 ± 9.5	<0.001
Waist circumference	90.44 ± 13.9	25.23 ± 3.5	NS
Systolic blood pressure (mmHg)	125.55 ± 13.5	123.10 ± 12.3	NS
Diastolic blood pressure (mmHg)	84.22 ± 7.0	81.93 ± 8.9	NS
Smoking (%)	29.4%	14.3%	NS
Hypertension (%)	64.71%	78.57%	NS
Dyslipidemia (%)	76.47%	64.29%	NS
Time since AA diagnosis (years)	21.07 ± 13.5	9.00 ± 4.9	0.11
SALT	99.00 ± 2.9	98.39 ± 4.2	NS
Glucose (mg/dL)	92.12 ± 8.6	88.21 ± 9.9	NS
Albumin (mg/dL)	4.31 ± 0.2	4.50 ± 0.3	NS
Cholesterol (mg/dL)	214.47 ± 47.6	193.71 ± 38.4	NS
HDL (mg/dL)	68.29 ± 29.7	59.71 ± 15.3	NS
LDL (mg/dL)	122.71 ± 32.6	119.50 ± 31.4	NS
Triglycerides (mg/dL)	115.94 ± 60.3	70.43 ± 23.3	0.017
AST (U/L)	25.24 ± 19.8	22.29 ± 8.6	NS
ALT (U/L)	28.29 ± 27.6	19.14 ± 8.1	NS
GGT (U/L)	33.35 ± 27.2	21.64 ± 14.5	NS
Alkaline phosphatase (U/L)	67.18 ± 20.4	74.93 ± 23.4	NS
CRP (mg/dL)	2.54 ± 2.4	2.35 ± 2.4	NS
HbA1-c (%)	5.49 ± 0.3	5.23 ± 0.1	0.003
Neutrophils (×10^3^/mL)	4.20 ± 1.3	3.58 ± 1.5	NS
Lymphocytes (×10^3^ mL)	2.01 ± 0.7	2.55 ± 0.7	0.021
Platelets (×10^3^/mL)	236.76 ± 48.9	253.07 ± 64.0	NS
NLR	2.23 ± 0.8	1.41 ± 0.5	0.004
SII	542.64 ± 258.4	367.62 ± 177.2	NS
TyG	8.46 ± 0.5	7.99 ± 0.4	0.013

SALT, Severity of Alopecia Tool; CRP, C-reactive protein; NLR, neutrophil/lymphocyte ratio; SII, systemic inflammatory index; TyG, triglyceride–glucose index; NS, non-significant. Data expressed as % (qualitative variables) or as mean ± standard deviation (quantitative variables).

## Data Availability

Dataset available on request from the authors.
